# mSphere of Influence: Host Metabolism Is an Integral Part of the Immune Response to Infectious Diseases

**DOI:** 10.1128/mSphere.00247-21

**Published:** 2021-07-07

**Authors:** Jeffrey M. Collins

**Affiliations:** a Division of Infectious Diseases, Department of Medicine, Emory University School of Medicine, Atlanta, Georgia, USA

**Keywords:** immunity, inflammation, metabolic modeling

## Abstract

Jeff Collins is a physician scientist in the Division of Infectious Diseases at Emory University focused on using metabolomics and systems biology to better understand the pathophysiology of tuberculosis disease, identify new biomarkers, and elucidate targets for host-directed therapeutics. In this mSphere of Influence article, he reflects on how the paper “Succinate is an inflammatory signal that induces IL-1β through HIF-1α” by Tannahill et al. (G. M. Tannahill, A. M. Curtis, J. Adamik, E. M. Palsson-McDermott, et al., Nature 496:238–242, 2013, https://doi.org/10.1038/nature11986) influenced him by highlighting the intersection between metabolism and the host response to infectious diseases.

## COMMENTARY

At a research meeting during my fellowship in infectious diseases, one of my mentors said, “At the end of your fellowship you will be well positioned for a career studying immunometabolism.” Initially, I found this statement perplexing. While my research focused on performing metabolomics analyses on plasma samples from patients with tuberculosis (TB) disease, I knew very little about the way metabolism and metabolites interact with the immune system. Like many scientists working in the application of “-omics” technologies to translational research, I envisioned the primary output of my work would be biomarker discovery, which is desperately needed to develop improved TB diagnostics. What better way to discover biomarkers of a disease state than to profile tens of thousands of small molecules in the biofluids of those affected? However, as the field of metabolomics has advanced, it has become increasingly clear that biomarker discovery is just one of many ways that studying the metabolome can enhance our knowledge of human disease. As demonstrated by the paper “Succinate is an inflammatory signal that induces IL-1β through HIF-1α” by Gillian Tannahill et al., metabolic remodeling of immune cells can have far-reaching effects on the host immune response (1).

Prior work indicated that lipopolysaccharide (LPS) activation of monocytes resulted in a switch in cellular energy metabolism from oxidative phosphorylation to glycolysis. The authors first observed that using 2-deoxyglucose to inhibit the shift to glycolysis impaired production of the proinflammatory cytokine interleukin-1β (IL-1β) but not other proinflammatory cytokines in macrophages activated by Bordetella pertussis and mice treated with LPS (1). They went on to use untargeted metabolomics to show that as activated macrophages switched to glycolysis as their primary energy source and citric acid cycle activity decreased, intermediates from the citric acid cycle, including succinate, fumarate, and malate, accumulated (1). Concentrations of succinate were highly correlated with expression of *Il1b* in mice and macrophages after LPS activation, an effect that was enhanced with addition of a cell-permeative form of succinate, suggesting succinate itself was driving the proinflammatory signaling cascade. Finally, the authors used mouse bone marrow-derived macrophages deficient in the transcription factor hypoxia-inducible factor-1α (HIF-1α) to show that increased *Il1b* transcription was dependent upon succinate interacting with and stabilizing HIF-1α (1). Thus, they demonstrated the metabolic remodeling of macrophages was not the product of an inflammatory signal. It *was* the signal.

The study by Tannahill et al. showed me that metabolites are an integral part of molecular networks that govern the host response to infectious diseases. Combining traditional immunologic studies with studies of metabolism is therefore critical to understand how humans do or do not control pathogens such as Mycobacterium tuberculosis. This article inspired me to expand my work in metabolomics to not only identify disease biomarkers but also gain new insights about the pathophysiology of TB disease. Studies in TB immunometabolism published to date indicate the metabolic remodeling described by Tannahill and colleagues is highly relevant to TB disease pathogenesis (2, 3). However, there is much to learn about how metabolites interact with other components of the immune system in the context of TB infection and disease and how this contributes to differential host outcomes. If we are to more fully understand the clinical heterogeneity observed in TB, we must improve our understanding of the metabolic contributors to immunity and immune evasion.

Following the publication by Tannahill and colleagues, others have contributed to understanding the vast number of molecular networks involved in generating protection from infectious diseases. In the paper “Metabolic phenotypes of response to vaccination in humans,” Li et al. captured the extensive redundancies and interconnections between metabolic signaling, transcriptional signaling, and immunogenicity following vaccination with attenuated varicella-zoster virus (4). The authors found that widespread perturbation of the plasma metabolome occurred almost immediately following vaccination. Metabolic responses 1 day after vaccination were strongly associated with transcriptional responses on day 3 as well as antibody- and cell-mediated immune responses weeks later. By day 7 after vaccination, the metabolome had largely returned to its prevaccination state (4). Thus, the authors showed that metabolic networks were an early and active part of the immune response resulting in protective immunity.

However, while the work by Li and colleagues gave us an idea of the sheer magnitude of the interconnections between metabolism and the immune response to pathogens, it also highlighted many of the challenges we face going forward. The field of metabolomics has advanced rapidly over the last 10 years, and it is now possible to routinely measure over 20,000 mass features in a single biospecimen. Combining such high-dimensional data with other systems data from the transcriptome, proteome, and microbiome as well as traditional measures of immunity such as immune cell subsets, cytokines, and antibody responses presents an enormous analytic challenge. Advancing the field of immunometabolism will therefore require substantial investment in computational approaches to better understand the many connections that exist between metabolism and immunity, elucidate their functional significance, and discover new targets for host-directed therapies that modulate inflammation.

As I move forward in my career as a translational TB researcher, I look forward to overcoming some of these challenges to gain a deeper understanding of how metabolism impacts the extraordinary heterogeneity in human outcomes following infection with M. tuberculosis. Recent technical advances in analytic chemistry and computational biology have made understanding the role of metabolism in human health and disease more accessible than ever before (5). As Tannahill and colleagues demonstrated in their seminal paper, studying metabolism is critical to more fully understand the human response to infectious diseases.
